# Beneficial Effects on Arterial Stiffness and Pulse-Wave Reflection of Combined Enalapril and Candesartan in Chronic Kidney Disease - A Randomized Trial

**DOI:** 10.1371/journal.pone.0041757

**Published:** 2012-07-31

**Authors:** Marie Frimodt-Møller, Anne-Lise Kamper, Svend Strandgaard, Svend Kreiner, Arne Høj Nielsen

**Affiliations:** 1 Departments of Nephrology, Rigshospitalet, University of Copenhagen, Copenhagen, Denmark; 2 Herlev Hospital, Copenhagen, Denmark; 3 Department of Biostatistics, University of Copenhagen, Copenhagen, Denmark; University of Oxford, United Kingdom

## Abstract

**Background:**

Cardiovascular disease (CVD) is highly prevalent in patients with chronic kidney disease (CKD). Inhibition of the renin-angiotensinsystem (RAS) in hypertension causes differential effects on central and brachial blood pressure (BP), which has been translated into improved outcome. The objective was to examine if a more complete inhibition of RAS by combining an angiotensin converting enzyme inhibitor (ACEI) and an angiotensin receptor antagonist (ARB) compared to monotherapy has an additive effect on central BP and pulse-wave velocity (PWV), which are known markers of CVD.

**Methods:**

Sixty-seven CKD patients (mean GFR 30, range 13–59 ml/min/1.73 m^2^) participated in an open randomized study of 16 weeks of monotherapy with either enalapril or candesartan followed by 8 weeks of dual blockade aiming at a total dose of 16 mg candesartan and 20 mg enalapril o.d. Pulse-wave measurements were performed at week 0, 8, 16 and 24 by the SphygmoCor device.

**Results:**

Significant additive BP independent reductions were found after dual blockade in aortic PWV (−0.3 m/s, P<0.05) and in augmentation index (−2%, P<0.01) compared to monotherapy. Furthermore pulse pressure amplification was improved (P<0.05) and central systolic BP reduced (−6 mmHg, P<0.01).

**Conclusions:**

Dual blockade of the RAS resulted in an additive BP independent reduction in pulse-wave reflection and arterial stiffness compared to monotherapy in CKD patients.

**Trial Registration:**

Clinical trial.gov NCT00235287

## Introduction

Markers of arterial stiffness such as aortic pulse-wave velocity (PWV) and central blood pressure (BP) are known independent predictors of cardiovascular morbidity and mortality in chronic kidney disease (CKD) [1]–[3]. Inhibition of the renin-angiotensinsystem (RAS) with an angiotensin converting enzyme inhibitor (ACEI) or an angiotensin receptor blocker (ARB) has been shown to afford cardio-renal protection beyond the BP lowering effects 4–8. This may be due to preferential lowering of the central BP by the RAS blockers compared to other antihypertensives [9], [10]. Central BP, which is markedly influenced by vascular stiffness, has been found to be a better predictor of cardiovascular outcome than the conventional brachial BP [11]–[13]. Treatment with combinations of ACEI and ARB in full doses would expectedly lead to a more complete blockade of the RAS than can be obtained with either drug group. Such dual blockade has been demonstrated to have beneficial effects on arterial wave reflection and PWV in resistant hypertension [14], [15]. Surprisingly, in the recent ONTARGET study no beneficial effect of dual blockade on cardio-renal outcome was found in high risk cardiovascular patients [16]. Furthermore, in another recent observational study dual blockade did not reduce cardiovascular death in chronic hemodialysis patients [17].

In the present study it was investigated for the first time whether in CKD patients dual RAS blockade has an additive effect on central pressure waves and arterial stiffness evaluated by pulse-wave analysis (PWA) and PWV respectively, compared to mono RAS blockade, and whether these effects if present are BP independent.

## Methods

The protocol for this trial and supporting CONSORT checklist are available as supporting information; see Checklist S1 and Protocol S1.

### Study Population

Sixty-seven patients, all Caucasians, from the outpatient nephrology clinic, Herlev University Hospital, 52 men and 15 women, mean age 60 (range 31–75) were enrolled in this open randomised cross-over trial from September 2005 to September 2009. All patients gave informed consent and the study was approved by the Ethical Committee of Copenhagen County. The authors adhered to the Declaration of Helsinki and the study was monitored by the Good Clinical Practice (GCP) unit at Copenhagen University Hospitals, and was registered by EudraCT number 2005-001568-29 and in the public trial registry: www.clinicaltrials.gov, registration number NCT00235287.

The eligibility criteria for patients entering the study were pre-dialysis CKD with plasma creatinine between 150 and 350 µmol/l, plasma potassium below 5.6 mmol/l, systolic BP above 109 mmHg and age between 18 and 75 years. Patients with congestive heart failure (NYHA III-IV), chronic liver insufficiency, amputation of a limb or the presence of cardiac arrhythmia or a pacemaker were not included. None of the patients were to be treated with immunosuppressives, non-steroidal anti-inflammatory drugs, aldosterone antagonists or dual RAS blockade at the entry of the study.

Seventy-two per cent of the patients were treated with ACEI or ARB before enrolment and thus were known RAS blockade tolerant. Additionally, most were treated with furosemide and non ACEI/ARB antihypertensive therapy, which were continued during the trial. Demographic data and renal diagnoses are shown in table 1.

**Table 1:** Demographic data of the studied patients.

	total N = 67 (%)
Gender (f/m)	15/52 (22/78)
Age (years)	60±6
Previous cardiovascular events	16 (24)
Smoking	9 (13)
Diabetes mellitus	14 (21)
**Kidney disease**	
Nephrosclerosis	6 (9)
Polycystic kidney disease	11 (16)
Diabetic nephropathy	2 (3)
Chronic glomerulonephritis	6 (9)
Unknown	37 (55)
Other	5 (7)
**Pre-trial antihypertensive treatment**	
ACE-inhibitior	22 (33)
Angiotensin receptor blocker	26 (39)
No RAS-blocking agents	19 (28)
Beta-blocker	26 (39)
Diuretics	43 (64)
Calcium-channel-blocker	39 (58)

### Study Protocol

In order to ensure close balance of the numbers in each group at any time during the trial, block randomization was used [18]. In every block of 10 participants five would be allocated to each arm of the trial.

#### Mono therapy period

*Randomization of patients treated with either an ACEI or an ARB prior to the study* was carried out by drawing a closed envelope; to ensure that half of the patients had enalapril for the first 16 weeks and the other half had candesartan the first 16 weeks.

*Randomization of patients not treated with an ACEI or ARB prior to the study* was likewise carried out by drawing an envelope from a bag to ensure that half of the patients had enalapril in the first 8 weeks and candesartan in the following 8 weeks and the other half of the patients had candesartan in the first 8 weeks and enalapril in the following 8 weeks. By this means, tolerance to either drug was demonstrated in the patients not previously treated with RAS blockers before dual blockade.

**Figure 1:**
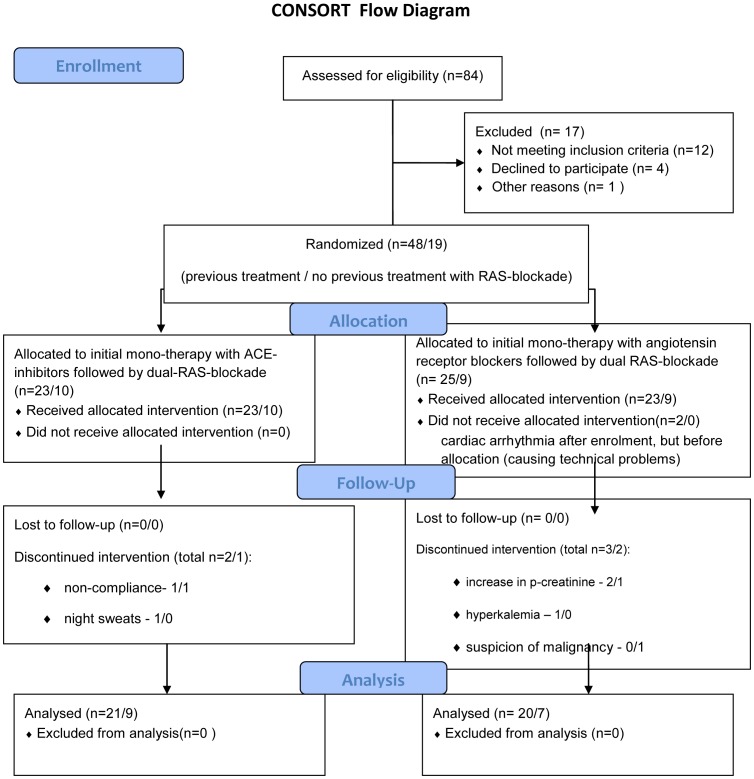
CONSORT Flowchart of the randomization in the study.

*In either of the four randomized patient groups*, doses were increased gradually over a period of 8 weeks from enalapril 5 mg to 20 mg, and from candesartan 4 mg to 16 mg, given once daily. All randomisations were carried out by the GCP-trained nurse staff members of the outpatient clinic without any conflict of interest in the trial. A flowchart of the randomized patients in the study is found in figure 1.

#### Dual blockade period

*All patients:* After 16 weeks of monotherapy with either enalapril or candesartan, the complementary drug was added in incremental doses over a period of 5 weeks, aiming at reaching a combination of enalapril 20 mg and candesartan 16 mg and maintaining this full dose dual blockade for an additionally 3 weeks. The therapeutic goal was a systolic/diastolic BP of 130/80 mmHg or below according to the K/DOQI-guidelines [19]. Additional antihypertensive treatment was simultaneously reduced, discontinued or added as needed.

During the 24 weeks study period, 10 control visits were planned: 4 visits in the laboratory, at which pulse-wave measurements were done and blood samples drawn, at the beginning of the trial and after 8, 16 and 24 weeks; 6 ‘clinical control’-visits in the outpatient clinic, at which BP was measured and blood samples drawn. All the visits were planned with an interval of 2–3 weeks throughout the study, so that 2 ‘clinical control’-visits were placed between two pulse-wave measurements. Glomerular filtration rate (GFR) was estimated at the first and last visit in the trial by the plasma clearance of ^51^Cr-EDTA as described below. Urinary albumin excretion/24 h was measured at each of the four visits of pulse-wave measurements.

#### Measurements of pulse-wave analysis and pulse-wave velocity

Measurements of PWA and PWV were performed using the SphygmoCor© device [20] (version 7.0, Atcor Medical, Sydney, Australia). All measurements were performed in the morning in the supine position after a minimum of 10 min rest in a quiet, temperature-controlled room. Patients were requested to be fasting and abstain from tea, coffee and smoking for 8 h and from alcohol for 24 h. Diabetic patients were allowed a light meal before examination. Study and other morning medication were to be taken 2 hours before measurements. The method of PWA and PWV measurement has been described in detail elsewhere [21]. Briefly, PWA was done with the use of a validated general transfer function [22]. The central pressure waveform was estimated based on radial pressure waveform recordings, calibrated to a brachial BP on the same arm. PWV was calculated based on the pulse transit time divided by the travel distance. The PWV of the ‘aortic’ segment (aortic PWV) was recorded between the femoral and carotid artery and the PWV of the ‘brachial’ segment (brachial PWV) was recorded between the radial and carotid artery. When determining the aortic PWV, the distance from the carotid recording site to the suprasternal notch was subtracted from the distance between the femoral recording site to the suprasternal notch [23]. When determining the brachial PWV, the distance from the carotid-suprasternal notch was subtracted from the distance between the radial-suprasternal notch.

The quality demands of PWA and PWV were followed as suggested by the manufacturer. This included visually acceptable pulse-wave recordings with variations in pulse height, diastole and pulse length ≤5% and the mean pulse height ≥80 mV as expressed by a quality index (%) provided by the software. A quality index ≥80% was accepted. In case of PWV, the time difference between the ECG-signal and the signal from the recording sites should have a SD ≤10% of the mean value. All measurements of PWA and PWV were made in duplicate.

*Management of hyperkalemia, excess rise in plasma creatinine and hypotension* have been described in details elsewhere [24].

### BP Measurement and Clinical Chemistry

The brachial BP used for calibration of PWA and all other brachial BPs were measured by use of a mercury sphygmomanometer after at least 10 min of supine rest. The mean of the last two out of three BP measurements were averaged and used for analysis. Plasma creatinine was analyzed using reagents from Vitros Chemistry 5.1, which are compatible with the IDMS method (isotope dilution mass spectrometry). Plasma potassium and other clinical chemistry parameters were measured using standard methods.

**Table 2:** Effects of dual RAS blockade on pulse-wave measurements and kidney function.

Intervention N = 57	start ACE-I/ARB	ACE-I/ARB	start dual blockade	end dual blockade
Brachial systolic BP (mmHg)	142±19	131±16	131±17	124±17***
Brachial diastolic BP (mmHg)	82±11	78±10	77±11	75±11 ^NS^
Brachial PP(mmHg)	60±18	53±13	54±16	50±15^ NS^
Central systolic BP (mmHg)	133±20	122±17	121±17	115±19**
Central diastolic BP(mmHg)	82±12	79±10	77±10	75±11^ NS^
Central PP(mmHg)	49±17	42±14	42±15	38±14^ NS^
TR (msec)	146±14	145±15	147±14	146±16^ NS^
Aix@HR75 (%)	21±10	20±11	19±10	17±13**
Brachial PWV (m/s)	9.9±1.4	9.6±1.7	9.5±1.3	9.5±1.7^ NS^
Aortic PWV (m/s)	9.6±2.8	9.2±2.5	9.0±2.7	8.7±2.8*
Heart rate (bpm)	62±11	62±11	61±11	61±10 ^NS^
ED (ms)	337±28	335±28	335±29	331±27^ NS^
P-creatinine (µmol/l)	216±57	228±70	232±67	239±78*
P-urea (mmol/l)	14±5	15±7	15±7	17±7***
P-potassium (mmol/l)	4.4±0.5	4.6±0.4	4.6±0.5	4.6±0.5^ NS^
GFR (Cr-EDTA, ml/min/1.73 m^2^)	**29.8 (13–60.1)**			23.9 (10–64)***
U-albumin excret. (µmol/24 h)	**2.4 (0.1–31.5)**	**2.3 (0.1–53.3)**	**1.5 (1.1–1.8)**	**0.9 (0.1–49.9)** ^ NS^

Blood pressure (BP), pulse pressure (PP), Time to Reflection (TR), heart rate adjusted Augmentation Index (AIxHR75), Pulse-wave velocity (PWV), Ejection Duration (ED).

P-value refers to statistical comparison between the end and start of dual blockade except for GFR which refers to comparison between start and end of study. *P<0.05, **P<0.01, ***P<0.001, ^NS^ = non-significant. Data in bold is geometric means with ranges in brackets.

### ^51^Cr-EDTA Clearance

Plasma clearance was calculated on the basis of plasma activity in four blood samples drawn at 20-min intervals in the fourth hour after injection [25]. In case of an expected GFR <21 ml/min, blood samples were drawn at 5 and 24 h after injection of ^51^Cr-EDTA [26].

**Figure 2:**
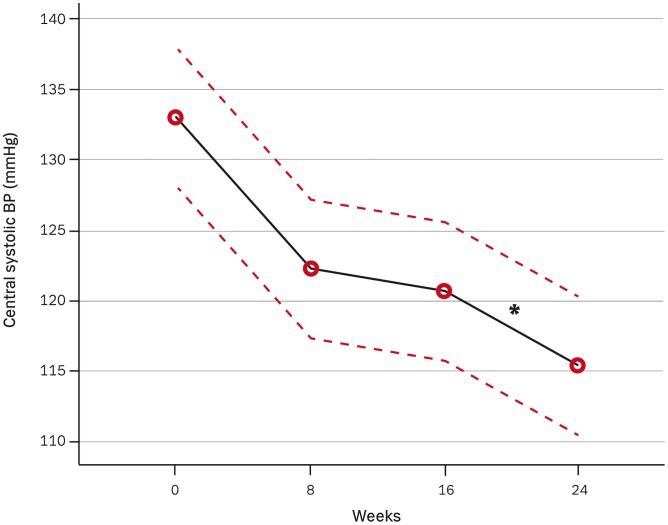
The effect of dual blockade compared to mono blockade on central systolic blood pressure with 95% confidence intervals as dotted lines. The number of measurement indicates 1. for baseline (mix of patients with and without previous treatment with either ACEI/ARB), 2. after 8 weeks of mono blockade with either ACEI/ARB, 3. after further 8 weeks of mono blockade and 4. after 8 weeks of dual blockade. * P<0.01.

### Arterial Hemodynamics

The pulse-wave is composed of an initial pulse-wave generated by left ventricular ejection and its reflection from the periphery. AIx@HR75 defined as the difference between the first and second systolic peaks expressed as a percentage of the pulse pressure adjusted to a heart rate of 75 beats/min- was taken to represent measures of arterial wave reflection [21]. Time to reflection (TR) was defined as the total travel time of the pulse-wave to the periphery and its return. Aortic PWV and brachial PWV are measures of central and muscular arterial stiffness, respectively [21] and were the pre-specified primary outcome measures. Pulse pressure (PP) amplification was calculated as the ratio of brachial PP/central PP [27]. Furthermore the following parameters were registered: central and brachial systolic and diastolic BP, left ventricular ejection duration (ED) and heart rate, which were the secondary outcome measures in addition to AIx@HR75, TR and PP-amplification.

**Figure 3:**
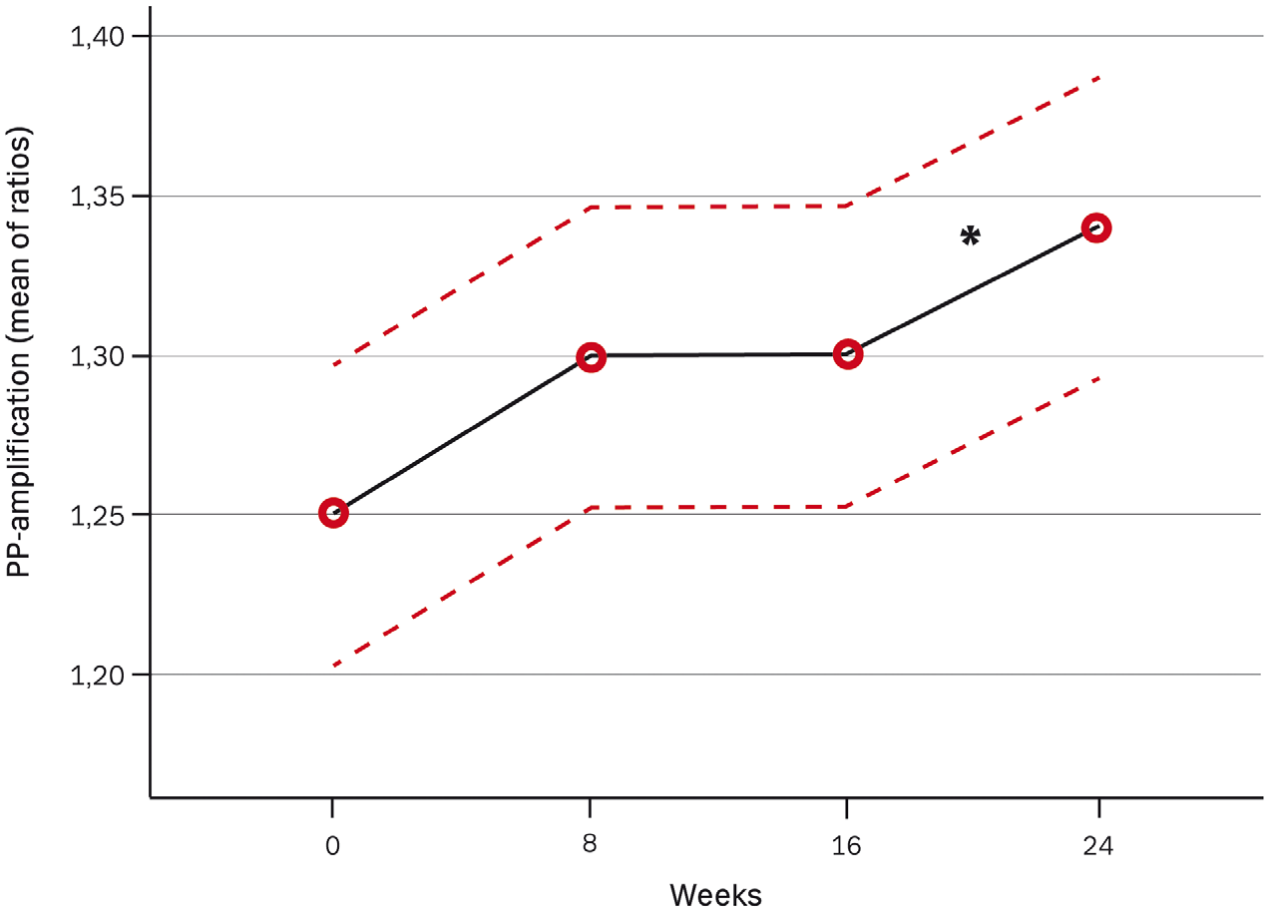
The effect of dual blockade compared with mono blockade on pulse-pressure-amplification with 95% confidence intervals as dotted lines. The number of measurement indicates 1. for baseline (mix of patients with and without previous treatment with either ACEI/ARB), 2. after 8 weeks of mono blockade with either ACEI/ARB, 3. after further 8 weeks of mono blockade and 4. after 8 weeks of dual blockade. * P<0.05.

### Statistics

The sample size was calculated using a two-sided paired sample t-test based on variations from previous data [28]. Twenty patients were needed without previous treatment with RAS-blockade, with previous treatment with ACE-I and with previous treatment with ARB, respectively. This sample size would give a 99% chance of detecting a difference of 1 m/s in aortic PWV and 80% in brachial PWV with an alpha-level of 5%.

**Figure 4:**
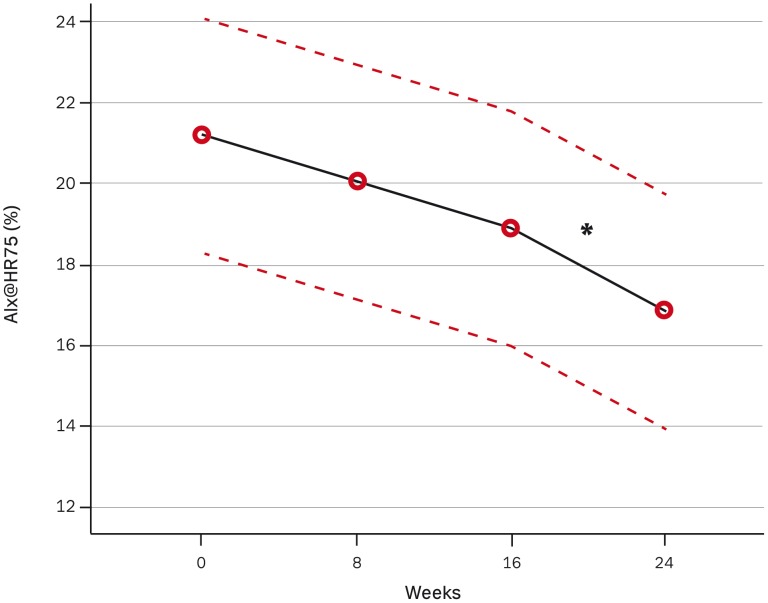
The effect of dual blockade compared with mono blockade on heart rate adjusted Augmentation Index (AIxHR75) with 95% confidence intervals as dotted lines. The number of measurement indicates 1. for baseline (mix of patients with and without previous treatment with either ACEI/ARB), 2. after 8 weeks of mono blockade with either ACEI/ARB, 3. after further 8 weeks of mono blockade and 4. after 8 weeks of dual blockade. * P<0.01.

The two groups with or without prior RAS-blockade were then randomized to four different treatment groups, which were compared for differences in demographic characteristics by analysis of variance. As no differences were found between groups demographic data are shown for all patients together. The effects of dual therapy and monotherapy on pulse-wave measurements were analyzed by general linear models for repeated measurements. As no differences were found between the four treatment arms data were pooled and further analyzed in total. We aimed to evaluate the effects of dual blockade on pulse-wave measurements and therefore based the statistical analysis on the 57 patients who completed dual blockade.

The effects of treatment on kidney function were evaluated by use of Student’s t-test for dependent data. During the analyses of repeated measurements, AIx@HR75, aortic PWV and brachial PWV were adjusted for diastolic BP, gender and age. Furthermore AIx@HR75 was adjusted for body-height, aortic PWV, TR and ED in a similar way, like the PP-amplification ratio which was adjusted for heart rate and body-height. Data are presented as mean ± standard deviation (SD) unless otherwise stated. Due to skewed distribution urine albumin excretion and GFR were log-transformed before analysis and the geometrical mean presented with range in brackets. A P-value <0.05 was considered significant. Data were analyzed by use of a statistical computer program (SPSS, version 17).

## Results

Of the 67 randomized patients, 57 completed the trial. All pulse-wave measurements were within our quality standard as described in the methods section. The mean quality index was 95±6%.

### Pooling of Randomization Groups

No differences were found between randomization groups in demographic data or baseline-values of pulse-wave measurements and clinical chemistry parameters. No differences were detected between treatment arms in the response of dual- versus mono-RAS blockade. These data are therefore presented as pooled data (table 1 and 2). Treatment with ACEI, ARB or the absence of RAS-blocking treatment prior to the trial, did not influence the response of dual RAS therapy versus mono-therapy for any of the parameters.

### Blood Pressures

The effects of mono- and dual RAS-blockade on pulse-wave measurements and on kidney function are presented in table 2. Brachial and central systolic BP (figure 2) decreased significantly after start of combination treatment compared to monotherapy, but no further change was seen in brachial and central diastolic BP. Even though neither central nor brachial pulse pressure (PP) achieved a significant reduction, a significant increase (P = 0.02) on PP amplification was seen as illustrated in figure 3. This PP amplification changed from 1.26 at study entry to 1.30 and 1.30 after 8 and 16 weeks of monotherapy respectively and increased to 1.34 after dual blockade without any influence of height or heart rate.

### Pulse-wave Velocity and Augmentation Index

A significant additive reduction was observed in aortic PWV of 0.3 m/s after combined treatment compared to monotherapy corresponding to a difference of 3%. This was independent of diastolic BP, age and gender. No significant change was detected in brachial PWV (table 2). The heart rate adjusted augmentation index.

(AIx@HR75) decreased significantly during dual blockade versus monotherapy by 2% corresponding to a proportional difference of 11%. This was independent of diastolic BP, age, body-height and gender (figure 4). This change in AIx@HR75 was related to the change in brachial PWV (P = 0.009) and not related to changes in Time to reflection (TR), Ejection duration (ED) or aortic PWV. No additive change was seen in TR, heart rate or ED during dual-blockade.

### Change in Clinical Chemistry Parameters

There was a further increase in p-creatinine and p-urea after dual blockade compared to mono-blockade (table 2). No additive effects were seen in p-potassium or urinary albumin excretion. There was a significant reduction seen in glomerular filtration rate (GFR) after dual blockade compared to baseline.

### Tolerance and Other Medication

Thirty-three (49%) of the patients failed to tolerate full dual blockade with enalapril and candesartan, and had to be given lower doses of one or both of the drugs, or in 10 cases withdrawn from the study [24]. Hence, 34 (51%) of the patients tolerated the full dual blockade. Eight patients were withdrawn during mono-therapy: 2 due to early technical difficulties with the pulse wave measurements, 3 due to adverse reactions (unacceptable increase in p-creatinine, intractable rise in p-potassium or night sweats), one patient due to suspicion of malignancy early in the trial and 2 patients due to non-compliance. Two patients were withdrawn during dual-therapy, both because of unacceptable increases in p-creatinine. There were no differences in other antihypertensive medicine between the mono-therapy and dual-therapy period. Notably, the frequency of increasing or prescribing diuretics due to hyperkalemia was the same for the two periods.

Transient hyperkalemia was frequently seen as previously described [24], but was evenly distributed between the two treatment regimens.

## Discussion

The main finding of this study is the BP independent reduction in arterial stiffness and pulse-wave reflection after combined treatment with enalapril and candesartan compared to monotherapy in patients with CKD. Furthermore pressure amplification was increased during dual blockade compared to monotherapy.

Aortic PWV is considered a direct measure of arterial stiffness [21] and is a strong independent predictor of all-cause and cardiovascular mortality and events in patients with ESRD [1], [29] and CKD [3]. We found a significant reduction in aortic PWV of 0.3 m/s during dual blockade independent of BP and corrected for age and gender. In contrast no significant change was seen in brachial PWV.

Pulse-waves are in most studies analyzed by three parameters: central PP, central systolic BP and AIx [21] of which all have shown independent predicting value of all-cause mortality in ESRD [2], [30]. In one study, for each increase of AIx of 10%, the relative mortality risk was increased by 1,51 [2]. We found a modest but significant reduction in the heart rate adjusted AIx (AIx@HR75) of 2% after dual blockade compared to a reduction during mono-blockade of 2% as well. Even after adjustment for known determinants of AIx as diastolic BP, height, gender and age [21], [31] the reduction in AIx@HR75 was still significant. This reduction in AIx@HR75 was related to the change in brachial PWV even though this change did not achieve statistical significance. This indicates that the reduction in the intensity of wave-reflection was the principal mechanism of the change in AIx@HR75 caused by a dilatation of medium-sized muscular arteries and thereby a reduced brachial PWV. This relationship has been emphasized by others [32].

Dual blockade significantly reduced brachial and central systolic BP on average by 7 and 6 mmHg respectively on top of a decrease during monotherapy of 11 and 12 mmHg respectively. A significant increase in PP-amplification was seen (figure 3), indicating a larger effect of dual blockade on central BP than on brachial BP. This was despite the lack of proven significant changes in central or brachial PP which were most likely caused by a statistical error type II due to a limited number of patients. A lower pressure amplification indicates a higher left ventricular afterload because of higher central PP [33]. Thus the reduction of PP amplification has prospectively been shown to be a strong independent predictor of all-cause and CV mortality in ESRD and superior to central PP [30]. RAS-blocking agents have previously been shown to cause increased pressure amplification. London et al. reported an increase in amplification ratio from 1.0 to 1.13 after treatment with perindopril in patients with ESRD for 12 months [34]. In hypertensive patients Dhakam et al. found an increase in PP amplification ratio from 1.38 to 1.42 after treatment with eprosartan for 6 weeks [35]. In our study, 8 weeks of dual blockade increased amplification ratio from 1.30 to 1.34.

No previous studies have been conducted on the effects of dual blockade on pulse-wave measurements in CKD patients. A study of 18 hypertensive patients with normal renal function treated with ACEI showed that add on valsartan reduced central BP more than brachial BP. A significant reduction in AIx of 13% was found after 2 weeks [14]. In contrast, the same group observed no further change in amplification in another study of 12 hypertensive patients with normal renal function, comparing the effects of valsartan, captopril and their combination [36]. Significant reductions of both the central and brachial BP were found, but to the same extent. Like us, they observed a BP independent additive reduction of AIx and aortic PWV during combination therapy compared to monotherapy. In a third study in 31 hypertensive patients with normal renal function, combining valsartan and perindopril did also further reduce PWV but without correction for BP [15].

Our patients had moderate to advanced CKD (stage 3–5) where inhibition of RAS can be a challenge due to loss of kidney function [37] and hyperkalemia [38]. Ten patients were withdrawn from the study including two during the dual blockade period. Patients were closely monitored and incidences such as hyperkalemia and unacceptable increases in p-creatinine [39] were carefully managed. Thus the full dose study medication of RAS blockers was reduced in 42% of the patients. We have previously reported on the feasibility of dual blockade in the first 47 patients of the study [24].

Reduction of BP is known to reduce arterial stiffness [32]. The fact that the decrease in aortic PWV and AIx@HR75 was independent of BP suggests that the decrease in arterial stiffness was caused by the direct effect of the drug on the vascular wall. The common feature of ACEI and ARB appears to be their dilating capacity on especially peripheral muscular arteries and their ability to reduce wave-reflections, as expressed by AIx [32]. Combination of ACEI and ARB might produce a more complete inhibition of RAS and enhance bradykinin accumulation resulting in increased endothelial NO production. Furthermore ANGII is known to cause cardiovascular remodelling and vascular hypertrophy, and it therefore seems likely that the action on vascular wall includes chronic inverse remodelling of the small arteries leading to improved viscoelastic properties. Because of the relatively short period of treatment in our study, the additive beneficial effects of dual blockade are probably caused by functional changes, but due to the BP independent findings structural changes cannot be excluded.

### Study Limitations

The cross-over design allowed for comparisons within the same patient. It would, however have been valuable with a control group treated with ACEI/ARB monotherapy parallel to the dual treatment period to rule out the time factor as a confounder. The period of dual blockade was deliberately not randomized, because of the crucial need for a cautious dose titration in each mono-therapy before start of dual blockade in these vulnerable patients, which necessitated the placement of the dual blockade treatment period in the end of the trial.

There was no statistical difference between randomization arms, which allowed for analysis of data pooled together. We cannot though, exclude the possibility that inter-group differences did exist, but were not found due to small numbers in each randomization group. However, we consider, that by pooling data more powerful results were achieved.

### Conclusion

In CKD patients, combined RAS blockade with enalapril and candesartan caused additive significant BP independent reductions in aortic PWV and AIx@HR75 compared to mono-therapy. Likewise, central systolic BP was reduced and PP-amplification was increased during dualblockade. As these are all independent predictors of cardiovascular mortality in CKD patients, the beneficial effects of dual RAS blockade found in this study might lead to a favorable effect on cardiovascular outcomes in CKD patients beyond that achieved by monotherapy.

## Supporting Information

Protocol S1 **Trial Protocol.**
(DOCX)

Checklist S1 **CONSORT Checklist.**
(DOC)
