# Supplementary material for: Beneficial Effects on Arterial Stiffness and Pulse-Wave Reflection of Combined Enalapril and Candesartan in Chronic Kidney Disease - A Randomized Trial
Source: PLoS One. 2012 Jul 31;7(7):e41757. doi: 10.1371/journal.pone.0041757 (PMC3409235; doi:10.1371/journal.pone.0041757)
Supplement: Protocol S1 — Trial Protocol. (DOCX) [file pone.0041757.s001.docx]

**Study protocol**

**Pulse-wave velocity and pulse-wave morphology in patients with chronic renal failure: The effect of blockade of the renin-angiotensin system**.

________________________________________________________________


Marie Frimodt-Møller, MD, research fellow *
 Arne Høj Nielsen, Professor, clinical associate professor, DMSc.*
Anne-Lise Kamper, chief physician, DMSc **
Svend Strandgaard, Professor, chief physician, DMSc .*


* Department of Nephrology, Copenhagen University Hospital in Herlev
** Department of Nephrology, Copenhagen University Hospital, Rigshospitalet.
Denmark.

**Table of Contents:**1. Project Leader and the investigator's signature ………………………………………..4
2. Layman Summary ……………………………………………………………………. 5
3. Summary ……………………………………………………………………………….6
4. General Information …………………………………………………………………...7

4.1 Project group
 4.2 Collaborators
 4.3 GCP-monitoring
 4.4 Locations

5. Description of project …………………………………………………………………..8
 5.1 Purpose
 5.2 Effect Parameters
 5.3 Background
 5.4 Methods
 5.5 Design
 5.6 Patient Population
 5.7 Inclusion Criteria
 5.8 Exclusion Criteria
 5.9 Criteria for withdrawal
 5.10 Demographic variables
 5.11 Para-clinical variables
 5.12 Practical performance

5.13 Medicine of the study
 5.14 Statistics

6. Quality control and quality assurance .……………………………………………19
7. Ethical considerations .…………………………………………………………....19
 7.1 Project Impact and Benefits
 7.2 Risks and disadvantages
 7.3 Summary
8. Funding …………………………………………………………………………...20
9. Patient information and consent …………………………………………………..21
10. Publication ………………………………………………………………………...22
11. Insurance…………………………………………………………………………...22
12. References……………………………………………………………………….....22

Appendix I: Flowchart of trial design
Ia: Flowchart for patients with prior ACE-I/AIIA-treatment
Ib: Flowchart for patients without prior ACE-I/AIIA-treatment

Appendix II: Patient material for disclosure: …………………………………..25
IIa: Patient Information - general information
IIb: Patient information - about the project and its progress
IIc: Patient Information - about the practical
IId: Patient Meeting Schedule
IIe: Consent Statement 1
IIf: Consent Statement 2
IIg: “Before you decide”.

Appendix III: Registration Form for demographic variables …….……………..35

Appendix IV: Registration Form for Biochemistry…………………….………..36
IVa: Registration Form for Biochemistry at the start of project
IVb: Registration Form by measurements and controls

Appendix V: Copy of notification to the Data Protection Agency and the Medicines Agency

Appendix VI: Copy of diploma from medical school

Appendix VII: Copy of medical authorization

Appendix VIII: Curriculum Vitae

1. **Project leader and investigator's signature**

The undersigned, hereby clinically responsible, is responsible for the described experiments carried out in accordance with protocol, GCP and applicable law.


... ... .... ... ... ... ... ... ... ... ... ... ... ... ... ... ... ... ... ... ... ... ... ... ... ... ... ... ... ... ....
Date Arne Høj Nielsen, clinically responsible


The undersigned sponsor and investigator are thus responsible for the described experiments carried out in accordance with protocol, GCP and applicable law.


... ... .... ... ... ... ... ... ... ... ... ... ... ... ... ... ... ... ... ... ... ... ... ... ... ... ... ... ... ... ....
Date Marie Frimodt-Møller, sponsor and investigator.

2. Layman Summary

Patients with chronic kidney disease have an increased risk of cardiac and vascular disease. It is shown that this is partly due to patients' arteries become stiffer than normal. The more rigid arteries become all the more change of heart beats (pulse-wave) in the arteries. This pulse-wave can be investigated with a newly developed apparatus - known as applanation tonometry. By placing a small pencil-shaped sensor on an artery with gentle pressure, it is possible by computer technology to reproduce the pulse-wave on the computer screen for further analysis. It is shown that changes in this pulse-wave appearance and velocity reflect changes in blood vessels. The method is tested both internationally and by our own group.

There is substantial evidence that the blockade of the so-called renin-angiotensin system in blood pressure lowering drugs of the types of ACE inhibitors or angiotensin receptor antagonists have a beneficial effect on this arterial stiffness and thus also on the incidence of cardiovascular disease and mortality. These two types of medications are used already widely in the treatment of patients with chronic kidney disease. Besides, that the medicines are blood pressure-lowering medication, it is known in many cases to inhibit the development of kidney disease.

Evidence suggests that by combining ACE inhibitors and angiotensin receptor antagonists an additional beneficial effect is obtained on the vascular system in addition to the effect of treatment with each drug. This is studied in several groups of patients, but the effect on arterial stiffness in kidney patients is only sparsely examined.

We want to examine whether treatment with a combination of an ACE inhibitor and an angiotensin receptor antagonist improves the treatment of cardiac and vascular disease in kidney patients more than treatment with each drug separately. We want to evaluate this by use of the aforementioned applanationtonometry where we measure arterial stiffness. We use the medicine Enalapril, an ACE inhibitor and Candesartan, an angiotensin receptor antagonist.

The study is conducted in 60 patients with chronic kidney disease. The experiment extends over 3 periods of approx. 8 weeks. Patients will be randomized to which treatment they will get in each period. Thus, patients in the first and second period will be treated with either Enalapril or Candesartan and in the third period will be in combination therapy with both Enalapril and Candesartan. There will be performed pulse-wave measurements at baseline and after each period, a total of 4 times. Additionally, there will be two visits during each period for blood sample collection and blood pressure measurement, a total of 6 appearances.

Participants in the project will also have an examination of the kidney function performed before and after the trial. The kidney function is measured by the so-called Chromium-EDTA clearance, by injecting a small amount of radioactive substance into a vein and subsequent blood sampling. This involves an amount of ionizing radiation equal to one day's background radiation.

Time and blood sampling may be perceived as a possible disadvantage for the patient. Applanationtonometry and blood pressure measurement are not invasive procedures and poses no security risk. Furthermore, treatment with the mentioned drugs could cause side effects, which usually is mild and transient. These are usually dry cough and, rarely, nausea, dizziness and headache and increased levels of potassium salt in the blood. If unacceptable side affects the patient will be excluded from the trial.
The project's patient population is a high risk group in terms of primary cardiovascular morbidity and mortality. ACE-I and AIIA has in several studies been shown to reduce this morbidity and mortality. For each patient one must expect a neutral effect of participation in the study. The advantage for the group of patients as a whole, however, is to obtain more knowledge about disease and treatment options for renal patients.
In summary, the advantages and disadvantages for the overall study group are assessed to balance.

General procedures for quality control and quality assurance will be followed in accordance with ICH GCP Guidelines. Data will be monitored by the University GCP unit.
The project is sponsored by private foundations. There are no ties to the pharmaceutical industry.

**3. Summary**
Title:

Pulse Wave Velocity and pulse-wave morphology in patients with chronic
renal failure: The effect of blockade of the renin-angiotensin system.

Purpose:

To evaluate the effect of dual blockade of renin-angiotensin system with ACE
inhibitors (enalapril) and angiotensin II antagonists (candesartan) on
pulse-wave morphology and pulse-wave velocity in patients with chronic renal insufficiency evaluated with the applanationtonometry method. Primary endpoint is
pulse wave velocity (PWV). Secondary efficacy parameters include Augmentation Index.

Population:

60 patients with chronic nephropathy. All patients followed in the Nephrology Outpatient Clinic, Copenhagen University Hospital in Herlev.

Practical performance:

The method used in this project is applanationtonometry. In this, a pencil shaped tonometer is placed with a light touch on the skin over the radial artery, femoral artery and carotid artery. With the recorded surface derived pulse-wave recordings the pulse-wave can be further analyzed. Pulse-wave analysis is carried out with reference to 1) velocity and 2) morphology.
The patient population is divided into 2 groups: one group treated with either ACE-I
or AIIA before baseline and another group without prior treatment with
ACE-I or AIIA. The trial included 3 treatment periods of 8
weeks. Patients are prior to the baseline randomized to either treatment with Enalapril or Candesartan in the two periods and a combination treatment with both drugs in
the third period. Applanationtonometry is performed before baseline and after each period, a total of 4 times. In each treatment period 2 visits are placed in order to control blood pressure and blood sampling, a total of 6 visits. Before and after
the trial, a Cr-EDTA clearance is performed to measure renal function. In addition, the patient is delivering a 24-hrs urine collection in connection with applanationtonometry measurements.

The following is recorded for each patient: sex, age, weight, height, blood pressure, pulse, Cr-EDTA clearance, renal diagnosis, date of diagnosis, other diagnoses,
medication use, daily tobacco consumption.
The following blood samples taken at baseline for analysis of: b-hemoglobin, b-
leukocytes, platelets, p-bilirubin, ALT-p, p-alkaline phosphatase, hsC-reactive
protein, p-creatinine, BUN, p-sodium, potassium, p-bicarbonate, b-glucose, p-
albumin, p-cholesterol, p-triglyceride, p-HDL, p­-LDL, p-urate, p-parathyroid hormone
and p-homocysteine.

The following blood samples taken at other measurements and control visits: b-hemoglobin, p-creatinine, BUN, p-albumin, p-potassium and sodium.

Analysis:

The recorded pulse-wave measurements undergo a mathematical analysis using a
computer program in order to characterize the pulse-wave and
pulse-wave velocities. Data will be statistically processed with the help of a
professional statistician.

**4. General Information**

4.1 PROJECT GROUP

MD, research fellow Marie Frimodt-Møller is the investigator and sponsor
Department of Nephrology
Copenhagen University Hospital in Herlev
Herlev Ringvej 75
2730 Herlev
Tel. 4488 4878, email: marfri01@herlevhosp.kbhamt.dk

Professor, MD, clinical associate professor, MD. Arne Høj Nielsen is clinically responsible.
Department of Nephrology
Copenhagen University Hospital in Herlev
Herlev Ringvej 75
2730 Herlev
Tel. 44884488 - pager 82277

MD, DMSc. Anne-Lise Kamper is co-supervisor
Professor, MD, DMSc. Svend Strandgaard is co-supervisor

4.2 PARTNERS

Bioanalyst Bodil Hellstrøm
Laboratory of Nephrology
Copenhagen University Hospital in Herlev
Herlev Ringvej 75
2730 Herlev
Tel. 4488 4488
Agreed task: Blood sampling and performance of blood analysis. Furthermore assistance in pulse-wave measurements.

Nurse Kirsten Bramsen
Department of Clinical Physiology and Nuclear Medicine
Copenhagen University Hospital in Herlev
Herlev Ringvej 75
2730 Herlev
Tel. 4488 4488
Agreed task: the performance of chromium-EDTA clearance

Professor Svend Kreiner
Department of Biostatistics
University of Copenhagen
Blegdamsvej 3
2200 Copenhagen N
Tel. 3532 7597

4.3 MONITORING

GCP-coordinator, MSc. Ellen Due

Copenhagen University Hospital GCP-unit
County Hospital in Gentofte, opg.15, 2nd
Niels Andersensvej 65
2900 Hellerup
Tel. 3977 7347

4.2 SITES AND TIMEFRAME

Department of Nephrology

Copenhagen University Hospital in Herlev
Herlev Ringvej 75
2730 Herlev

Timeframe: September 2005 to September 2006

**5. Description of project**

5.1 PURPOSE

To assess the effect of dual blockade of renin-angiotensin system with ACE inhibitors (ACE-I) and angiotensin II antagonists (AIIA) on pulse-wave morphology and pulse-wave velocity (PWV) in patients with chronic renal failure assessed with the applanationtonometry method. Furthermore, a comparison of the effect of single blockade respectively ACE-I and AIIA is wanted.
The hypothesis is that dual blockade of renin-angiotensin system leads to a greater reduction of arterial stiffness than single therapy alone. We expect no difference in treatment effect between ACE-I and AIIA.

5.2 OUTPUT PARAMETERS

Primary endpoint is PWV, where smallest clinically relevant difference is 1 m / s. Secondary efficacy parameters Augmentation Index (AIx), blood pressure, Buckbergs ratio, heart rate, Time to reflection (TR), ejection duration (ED), Pulse Pressure (PP), ΔGFR, proteinuria, and other blood analysis answers.
5.3 BACKGROUND

The number of patients treated for chronic renal failure is increasing rapidly throughout the Western world. This is largely because we now offer dialysis to all with a dialysis need, and in contrast to earlier also in elderly patients with concurrent diseases. The number of patients on chronic dialysis treatment in Denmark has more than doubled over the past 10 years from 1177 in 1993 to 2429 at the end of 2003 (1). Only a small proportion of these patients can be treated with renal transplantation.

Patients with chronic kidney disease have an increased cardiovascular morbidity and mortality. Chronic dialysis patients have thus 10 to 20 times higher risk of cardiovascular death compared with the general population (2) and a mean survival of only about 3 years. The vast majority die from cardiovascular disease. The uraemic vascular disease differs markedly from the ordinary atherosclerotic vascular disease, with media sclerosis and media calcification is far more prominent, it is therefore of great interest to obtain more knowledge about the vascular dysfunction and disease in these conditions and any opportunities for intervention.

Numerous studies have shown that blockade of the renin-angiotensin system with either angiotensin converting enzyme inhibitors (ACE-I) or angiotensin receptorantagonists (AIIA) has beneficial effects in patients with heart failure (3). In essential hypertension it has been shown that this blockade resulted in regression of the abnormal vascular structure (4). In hypertension with left ventricular hypertrophy, an improved survival and regression of hypertrophy has been demonstrated by this treatment (5), as well as preventing repeated stroke (6-8). There is substantial evidence that the blockade of the renin-angiotensin system can affect cardiovascular morbidity and mortality in a positive direction.
ACE-I and AIIA has widespread use in the predialytic phase of chronic nephropathy where the treatment primarily by an antiproteinuric effect reduces the progression of uremia in type-1-diabetes with nephropathy (9,10), type-2-diabetes with hypertension (11) and not diabetic nephropathy (12-14).
Combination therapy with ACE-I and AIIA has been used in several large studies in which patients with heart failure in two studies demonstrated a better survival for selected patients (15.16) but in another study no additive therapeutic effect (17). In type-2-diabetes, an additive reduction in blood pressure and microalbuminuria has been shown (18) and an additive delay of deterioration of renal function has been shown in non diabetic chronic nephropathy (19). The rationale for combination therapy with ACE-I and AIIA is the potential of obtaining a more complete blockade of the renin-angiotensin system. AIIA is pharmacologically competitive reversible antagonists. The inhibition is thus reversible and can be counteracted by an increased concentration of angiotensin II. In combination with ACE-I the level of angiotensin II is reduced, thereby increasing the efficiency of the AIIA.

5.4 METHODS

The arterial system is in this project evaluated by applanationtonometry, which is a noninvasive method for analysis of the pulse-wave morphology and velocity (20-22). By using the so-called transfer function, the method can also estimate central blood pressure and pulse-wave morphology in the ascending aorta. This has been validated in invasive studies in patients with normal renal function (23,24). Changes in these central and peripheral parameters reflect changes in vascular structure and function compatible with changes in arterial stiffness.
Several studies have shown a good reproducibility of the method with acceptable inter-and intra-observer variation (25-29). In a previous study with 23 healthy individuals, we have shown that the measurements are influenced by smoking and food intake, and on which side of the body measurements are made (29).
Increasing attention is paid to arterial stiffness, including pulse wave velocity and pulse wave reflection, as cardiovascular risk markers. There is thus found an increased stiffness in patients with coronary artery disease (30), hypertension (31), and diabetes mellitus (32). Also in patients with chronic kidney disease, abnormally elevated pulse wave velocities and pulse wave reflections in dialysis patients have been found as predictors of cardiovascular death (33, 34). Whether this is true in patients with predialytic chronic kidney disease is poorly described.

The possibility of using applanationtonometry to get information about the central hemodynamics offers new perspectives compared to the conventional brachial measured blood pressure.
The REASON study found a significantly greater reduction of central systolic blood pressure and pulse pressure during treatment with the ACE inhibitor perindopril compared with beta-blocker atenolol, despite an equally large reduction of the peripherally measured blood pressure (35). Several studies have shown that either ACE-I or AIIA reduces the arterial stiffness evaluated by the applanationtonometry method (35,36). Also in patients with chronic nephropathy, a beneficial effect of both ACE-I and AIIAs has been found on arterial stiffness (37.38). The effect of combination therapy on arterial stiffness in kidney patients is only sparsely described (39), as well as a comparison of the two drugs in monotherapy.

It can therefore be summarized that applanationtonometry is a promising validated and reproducible method to study changes in the peripheral and central hemodynamic, including arterial stiffness. There is evidence that arterial stiffness, evaluated by this method, is a strong predictor of cardiovascular death in patients with chronic kidney disease. Blocking the renin-angiotensin system with ACE-I or AIIA has been shown to reduce this arterial stiffness and represents the mainstay of treatment in many cardiovascular and renal disorders. Evidence suggests that combination therapy gives an additive beneficial effect in several groups of patients, but the effect on arterial stiffness in kidney patients is only sparsely examined. A controlled clinical intervention study is therefore needed to investigate the effect of such dual blockade of the renin-angiotensin system in kidney patients. Positive additive effects could contribute to general recommendations regarding blockade of the renin-angiotensin system in patients with chronic kidney disease in an attempt to reduce the high cardiovascular morbidity and mortality.

5.5 DESIGN

Open randomized crossover trial in patients with non-dialysis chronic renal failure study:

• The effect of treatment with either ACE-I or AIIA on parameters of arterial stiffness assessed by the applanationtonometry method.
• The effect of treatment with both ACE-I and AIIA on parameters of arterial stiffness assessed by the applanationtonometry method.

The patient population is divided into 2 groups: one group with prior treatment with either ACE-I or AIIA (group I) and another group without prior treatment with either ACE-I or AIIA (group II). The study medication is as follows: the ACE inhibitor Enalapril and angiotensin receptor antagonist Candesartan. The study extends over three periods of 8 weeks. Patients in group I are randomized to receive either Enalapril in both the first and second periods or Candesartan in both the first and second periods. Patients in group II is randomized to either treatment with Enalapril or candesartan in the first period with a shift to the opposite drug in the second period.
Common to both groups is a double treatment with both Enalaprilat and Candesartan in the third period.
There will be carried out measurements at baseline and after the first, second and third period. Each study includes measurement by the applanationtonometry method where the tonometry is placed with gentle pressure towards the skin above the radial artery , thereby providing data for pulse wave analysis. Subsequently, measurements are performed equivalent to the radial, carotid and femoral arteries, thereby providing data for pulse wave velocity determination. The study design is shown in flow diagrams attached as Annex Ia + b.
There must be delivered 24 h urine collection before baseline and after every period. Chromium-EDTA clearance is carried out before and at the last visit in the trial.

*Patients receiving ACE-I or AIIA therapy prior to the trial.*
Pulse wave measurements are carried out at baseline and after each treatment period of 8 weeks, a total of 4 times. In addition comes 6 clinical control sessions and two appearances for the making of Chromium-EDTA clearance before and after the project.

Measurement I (start)
• At the first day of the study the patient meets fasting from midnight and without smoking, but with permission to drink water. Alcohol should not be taken for 24 hours before study start. The patient waits to take his medication until after the study. For patients with diabetes, however, a light breakfast is consumed at home and the usual medications for diabetes, all medicines taken after the study. Investigations commenced approx. at. 9:00 after blood sampling and then 15 min. rest in supine position. The studies are carried out in a quiet room with ambient temperature 20-22 degrees. A total of 2 measurements are carried out by the same investigator.
After the measurement the patient's habitual ACE-I or AIIA therapy is discontinued and patients are randomized to a scenario with either Enalapril in the first and second periods (ACE-I group) or treatment with Candesartan in the first and second period (AIIA group). Common to both randomization groups is a common dual blockade with both Enalapril and Candesartan in the third period.
The ACE-I group is started in tablet Enalapril 5 mg daily and is given tablets until the next appointment. AIIA group is started in tablet Candesartan 4 mg daily and are given tablets until next appointment. For women of childbearing age, the drugs will only be supplied in case of a negative pregnancy test.

The patient must before start of treatment have had a Chromium-EDTA clearance performed as well as a 24 h urine collection, which is attempted planned for the day of measurement I.

Adjustment of medicine and clinical control Ia (after 2-3 weeks)
• Attendance in the morning for blood pressure measurement and blood sampling. Control of treatment. The AIIA-group is increased to a tablet of candesartan 8 mg daily and the ACE-I group is increased to a tablet Enalapril10 mg daily. Both groups were handed out tablets for next appointment.

Adjustment of medicine and clinical control Ib (after 5-6 weeks weeks)
• Attendance in the morning for blood pressure measurement and blood sampling. Control of treatment. The AIIA group increased to a tablet of candesartan 16 mg daily and the ACE-I group increased to a tablet Enalapril 20 mg daily. Both groups were handed out tablets for next appointment.

Measurement II (after approx. 8 weeks, 1^st^ period)
• The patient meets fasting from midnight and without prior smoking, but with permission to drink water. Alcohol should not be taken for 24 hours before study start. A 24 hour urine collection is delivered for analysis. The patient is taking the study medication approx. 2 hours before meeting time. Any other medication is to be taken after the measurements on that day. For patients with diabetes however, a light breakfast and the usual medications for diabetes and the study medication are consumed at home, all other medication is to be taken after the measurement that day. Investigations are started approx. at. 9:00 after blood sampling and then 15 min. rest in the supine position. The measurements are carried out in a quiet room with ambient temperature 20-22 degrees. A total of 2 measurements are performed by the same investigator.
After the measurement, the patients in the ACE-I group continue taking a tablet of Enalapril 20 mg daily and patients in the AIIA group continue taking tablet candesartan 16 mg daily. They are handed out tablets to the next appointment.

Adjustment of medicine and clinical control IIa (after 10-11 weeks)
• Attendance in the morning for blood pressure measurement and blood sampling. Control of treatment. Patients in the ACE-I group continue on tablet Enalapril 20 mg daily. The AIIA group continues on tablet candesartan 16 mg daily. They are handed out tablets to next appointment.

Adjustment and control IIb (after 13-14 weeks)
• Attendance in the morning for blood pressure measurement and blood sampling. Control of treatment. Patients in the ACE-I group continue on tablet Enalapril 20 mg daily. The AIIA group continues on tablet candesartan 16 mg daily. They are handed out tablets to next appointment.

Measurement III (after about 16 weeks, second period)
• The patient meets fasting from midnight and without prior smoking, but with permission to drink water. Alcohol should not be taken for 24 hours before study start. A 24 hour urine collection is delivered for analysis. The patient is taking the study medication approx. 2 hours before meeting time. Any other medication is to be taken after the measurements on that day. For patients with diabetes however, a light breakfast and the usual medications for diabetes and the study medication are consumed at home, all other medications is to be taken after the measurement that day. Investigations are started approx. at. 9:00 after blood sampling and then 15 min. rest in the supine position. The measurements are carried out in a quiet room with ambient temperature 20-22 degrees. A total of 2 measurements are performed by the same investigator.
After the measurement, the patients in the ACE-I group are supplemented with tablet candesartan 4 mg daily. Tablet Enalapril is continued in the same dose of 20 mg daily. Patients in the AIIA group is supplemented with Enalapril tablet 5 mg tablet daily and Candesartan is continued in the same dose of 16 mg daily. For both groups tablets are handed out to the next appointment.

Adjustment of medicine and clinical control IIIa (after 18-19 weeks)
• Attendance in the morning for blood pressure measurement and blood sampling. Control of treatment.
In the ACE-I group the dose of tablet candesartan is increased to 8 mg daily. Enalapril continues in the same dose of 20 mg daily. The AIIA group is increased to a tablet Enalapril 10 mg tablet daily and Candesartan continues in the same dose of 16 mg daily. They are handed out tablets to the next appointment.

Adjustment of medicine and clinical control IIIa (after 21-22 weeks)
• Attendance in the morning for blood pressure measurement and blood sampling. Control of treatment.
In the ACE-I group the dose of a tablet candesartan is increased to 16 mg daily. Enalapril continues in the same dose of 20 mg daily. The AIIA group is increased to a tablet Enalapril 20 mg tablet daily and Candesartan continues in the same dose of 16 mg daily. They are handed out tablets to the next appointment.

Measurement IV (after approx. 24 weeks, third period)
• The patient meets fasting from midnight and without prior smoking, but with permission to drink water. Alcohol should not be taken for 24 hours before study start. A 24 hour urine collection is delivered for analysis. The patient is taking the study medication approx. 2 hours before meeting time. Any other medication is to be taken after the measurements on that day. For patients with diabetes however, a light breakfast and the usual medications for diabetes and the study medication are consumed at home, all other medications is to be taken after the measurement that day. Investigations are started approx. at. 9:00 after blood sampling and then 15 min. rest in the supine position. The measurements are carried out in a quiet room with ambient temperature 20-22 degrees. A total of 2 measurements are performed by the same investigator.
After the measurement, the study medication is discontinued and the patient returns to his habitual medication. Eventually the patient is having a Chromium-EDTA clearance performed.

*Patients without prior ACE-I / AIIA therapy*Pulse wave measurements are carried out at baseline and after each treatment period of 8 weeks, a total of 4 times. In addition comes 6 clinical control sessions and two appearances for the making of Chromium-EDTA clearance before and after the project.

Measurement I (start)
• At the first day of the study the patient meets fasting from midnight and without smoking, but with permission to drink water. Alcohol should not be taken for 24 hours before study start. The patient waits to take his medication until after the study. For patients with diabetes, however, a light breakfast is consumed at home and the usual medications for diabetes, all medicines taken after the study. Investigations commenced approx. at. 9:00 after blood sampling and then 15 min. rest in supine position. The studies are carried out in a quiet room with ambient temperature 20-22 degrees. A total of 2 measurements are carried out by the same investigator.
After the measurement the patient is randomized to a scenario with either ACE-I treatment in the first period and switch to the AIIA treatment in the second period (group A) or a course of AIIA treatment in the first period and change to ACE-I in the second period (group B). Common to both randomization groups is a common dual blockade with both Enalapril and Candesartan in the third period.

Group A is started treatment with tablet Enalapril 5 mg daily. Group B is started treatment with tablet candesartan 4 mg daily. They are handed out tablets to next appointment. For women of childbearing age, the drugs will only be supplied in case of a negative pregnancy test.

The patient must before start of treatment have had a Chromium-EDTA clearance performed as well as a 24 h urine collection, which is attempted planned for the day of measurement I.

Adjustment of medicine and clinical control Ia (after 2-3 weeks)
• Attendance in the morning for blood pressure measurement and blood sampling. Control of treatment. Patients in group A is increased to tablet Enalapril 10 mg daily and patients in group B increased to tablet candesartan 8 mg daily. Both groups are handed out tablets for the next appointment.

Adjustment of medicine and clinical control Ib (after 5-6 weeks)
• Attendance in the morning for blood pressure measurement and blood sampling. Control of treatment. Patients in group A are increased to tablet Enalapril 20 mg daily and patients in group B are increased to tablet candesartan 16 mg daily. Both groups are handed out tablets for the next appointment.

Measurement II (after approx. 8 weeks 1^st^ period)
• The patient meets fasting from midnight and without prior smoking, but with permission to drink water. Alcohol should not be taken for 24 hours before study start. A 24 hour urine collection is delivered for analysis. The patient is taking the study medication approx. 2 hours before meeting time. Any other medication is to be taken after the measurements on that day. For patients with diabetes however, a light breakfast and the usual medications for diabetes and the study medication are consumed at home, all other medications is to be taken after the measurement that day. Investigations are started approx. at. 9:00 after blood sampling and then 15 min. rest in the supine position. The measurements are carried out in a quiet room with ambient temperature 20-22 degrees. A total of 2 measurements are performed by the same investigator.
After the measurement the patients in group A are changed to tablet Candesartan 4 mg and will no longer take Enalapril tablet. Patients in group B are changed to tablet Enalapril 5 mg daily and will no longer take tablet Candesartan. They are handed out tablets to the next appointment.

Adjustment of medicine and clinical control IIa (after 10-11 weeks)
• Attendance in the morning for blood pressure measurement and blood sampling. Control of treatment. Patients in group A are increased to tablet candesartan 8 mg daily. Patients in group B are increased to tablet Enalapril 10 mg daily. They are handed out tablets to the next appointment.

Adjustment of medicine and clinical control IIb (after 13-14 weeks)
• Attendance in the morning for blood pressure measurement and blood sampling. Control of treatment. Patients in group A are increased to tablet candesartan 16 mg daily. Patients in group B are increased to tablet Enalapril 20 mg daily. They are handed out tablets to the next appointment.

Measurement III (after about 16 weeks, second period)
• The patient meets fasting from midnight and without prior smoking, but with permission to drink water. Alcohol should not be taken for 24 hours before study start. A 24 hour urine collection is delivered for analysis. The patient is taking the study medication approx. 2 hours before meeting time. Any other medication is to be taken after the measurements on that day. For patients with diabetes however, a light breakfast and the usual medications for diabetes and the study medication are consumed at home, all other medications is to be taken after the measurement that day. Investigations are started approx. at. 9:00 after blood sampling and then 15 min. rest in the supine position. The measurements are carried out in a quiet room with ambient temperature 20-22 degrees. A total of 2 measurements are performed by the same investigator.
After the measurement the patients in group A are supplemented with tablet Enalapril 5 mg daily in addition to tablet candesartan 16 mg daily. Patients in group B are supplemented with tablet candesartan 4 mg daily in addition to tablet Enalapril 20 mg daily. They are handed out tablets to the next appointment.

Adjustment of medicine and clinical control IIIa (after 18-19 weeks)
• Attendance in the morning for blood pressure measurement and blood sampling. Control of treatment.
Patients in group A are increased to tablet Enalapril 10 mg daily and continued on the same dose of tablet Candesartan. Patients in group B are increased to tablet candesartan 8 mg daily and continued on the same dose of Enalapril tablets. They are handed out tablets to the next appointment.

Adjustment of medicine and clinical control IIIa (after 21-22 weeks)
• Attendance in the morning for blood pressure measurement and blood sampling. Control of treatment.
Patients in group A are increased to tablet Enalapril 20 mg daily and continued on the same dose of tablet Candesartan. Patients in group B are increased to tablet candesartan 16 mg daily and continued on the same dose of Enalapril tablets. They are handed out tablets to the next appointment.

Measurement IV (after approx. 24 weeks, third period)
• The patient meets fasting from midnight and without prior smoking, but with permission to drink water. Alcohol should not be taken for 24 hours before study start. A 24 hour urine collection is delivered for analysis. The patient is taking the study medication approx. 2 hours before meeting time. Any other medication is to be taken after the measurements on that day. For patients with diabetes however, a light breakfast and the usual medications for diabetes and the study medication are consumed at home, all other medications is to be taken after the measurement that day. Investigations are started approx. at. 9:00 after blood sampling and then 15 min. rest in the supine position. The measurements are carried out in a quiet room with ambient temperature 20-22 degrees. A total of 2 measurements are performed by the same investigator.
After the measurement, the study medication is discontinued and the patient returns to his habitual medication. Eventually the patient is having a Chromium-EDTA clearance performed.

5.6 PATIENT POPULATION

Potential participants are patients with chronic nephropathy treated in Nephrology Department, Copenhagen County Hospital in Herlev. The Patients are contacted in connection with a visit to the department and informed about the project. Interested patients meeting inclusion and exclusion criteria are included in the project. No fee is paid to study participants.

A total of 60 patients are scheduled to participate: 20 patients with prior ACE-I therapy, 20 patients with prior AIIA therapy and 20 patients without prior treatment with either ACE-I or AIIA at the entry of the study.

5.7 INCLUSION CRITERIA

The patient can be included in the project if the answer is YES to the following:
- Age between 18 and 75 years
- Have submitted voluntary written informed consent
- Have plasma creatinine between 150-350 µmol/l
- Systolic blood pressure> 110 mmHg
- Negative pregnancy test in fertile women

5.8 EXCLUSION CRITERIA

The patient may not be included in the project if the answer is YES to at least one of the following:
- Treatment with both ACE-I and A-II-A at inclusion time
- Pregnancy or lactation
- Treatment with immunosuppressive, steroids or NSAIDs
- Severe chronic heart failure (NYHA III-IV)
- Chronic liver disease
- Suspected or confirmed renal artery stenosis
- Cardiac arrhythmia and / or implantable pacemaker
- AMI or cerebrovascular event within the last 3 months
- Allergy to ACE-I or AIIA
- Amputation of a limb or crural or femural amputation.
- Dementia or a mental condition that prevents understanding of study requirements
- Dialysis or transplantation
- Treatment with aldosterone antagonists
- Plasma-potassium> 5.5 mmol/l
- Other severe chronic non-renal disease

5.9 CRITERIA FOR WITHDRAWAL

Study participants will be excluded from the trial if the following conditions occur:

- Pregnancy
- Adjacent to other serious illness
- Unacceptable side effects such as angioneurotic edema
- Intractable hyperkalemia
- Cardiovascular event such as AMI or cerebral apoplexia
- Increase in p-creatinine of> 30% from baseline
- Request from the study participant concerning exclusion

Decision on the withdrawal of the study participant from the trial can be carried out by the investigator, the clinical supervisor and the two co-supervisors.

5.10 DEMOGRAFIS KE VARIABLE

The following is recorded for each patient: sex, age, weight, height, blood pressure, heart rate, Cr-EDTA clearance, renal diagnosis, date of diagnosis, if any other diagnoses, habitual medication, daily tobacco consumption. A case report form will be prepared for each patient comprehensive registration form for demographic and paraclinical variables (Annex IV-VI) and data from applanationtonometry measurements. Data will be regularly entered for statistical analysis using the computer. The patient's name and Social Security number will not be entered, but the patient will be assigned a number in chronological order.

5.11 PARACLINICAL VARIABLE

The gained data is processed in the apparatus via a mathematical analysis to characterize the pulse wave and pulse wave velocity.
The following blood samples are taken at baseline and at all pulse wave measurements on all patients: b-hemoglobin, b-leukocytes, b-platelets, p-bilirubin, p-ALT, p-alkaline phosphatase, hsC-reactive protein, p-creatinine, p- urea, p-sodium, p-potassium, p-bicarbonate, glucose, p-albumin, p-cholesterol, p-triglyceride, p-HDL, p-LDL, p-urate, p-parathyroid hormone and p-homocysteine.

The following blood samples are taken at each clinical control: b-hemoglobin, p-creatinine, p-BUN, p-albumin, p-potassium and sodium.

*For blood samples:*At each blood sample a total of 30-40 ml of blood is taken. The blood will be analyzed immediately in connection with the measurement. An extra glass of blood will be taken in case of the need of reanalysis of samples.

*24 h urine collection*:
At baseline and at each measurement a 24 h urine collection ís delivered for analysis of urinary albumin and urinary sodium. The urine will be analyzed immediately in connection with the investigation. An extra glass of urine will be taken in case of the need of reanalysis of the sample. A pregnancy test in fertile women will be performed on a urine sample prior to entry into the study.

*Chromium-EDTA clearance*:
The estimated GFR <20 ml/min: 24h clearance with blood 5 and 24 hours after injection of 3.5 MBq of 51-Cr-EDTA.
The estimated GFR> 20 ml/min: blood sampling after 180, 200, 220 and 240 min. after injection of 3.5 MBq 51-CR-EDTA.

*Procedure regarding data at project completion*
All data will be stored for a maximum of 15 years, after which they will be destroyed. Data is stored in Nephrology Section B, KAS Herlev, and among the members of the project group. Data will only be available to the project group.

5.12 PRACTICAL PERFORMANCE

Patients who are interested in participating in the study are informed about the project. The written patient information (Annex IA, IB and III) obtained and reviewed and a possibly. consent is issued.

A time schedule is agreed for Chromium-EDTA clearance, the measurements and the clinical controls.

Demographic data is recorded, possibly using the patient record.

*Randomization*Prior treatment with ACE-I/AIIA: Randomization is done by drawing lots from a bag in which there are 40 notes. Half of these are inflicted ACE-I treatment and the other half is applied AIIA therapy. Randomization for the group of patients without prior ACE-I/AIIA is done in the same way by lot from a bag, half of the 20 notes bearing the ACE-I in the first period and AIIA in the second period and the other half is applied AIIA in the first period, and ACE-I in the second period. The result of the randomization is recorded in the patient's Case Report Form.

*Measurement I-VI*The day before the measurements the patient collects urine in a dunk.
At the day of the first measurement the patient does not take his usual medications until after the measurement. In case of diabetes, the habitual diabetes medication is taken as usual, but all other medications are taken after the measurement. At the other measurements the study medication is taken 2 hours before meeting time, but all other medications taken after the measurement, except from antidiabetic medication. On arrival the patient is weighed. The patient brings 24 h urine collection.

At study start (baseline) blood sampling is taken in a peripheral vein for analysis of: b-hemoglobin, b-leukocytes, b-platelets, p-bilirubin, p-ALT, p-alkaline phosphatase, hsC-reactive protein, p-creatinine , p-urea, p-sodium, p-potassium, p-bicarbonate, glucose, p-albumin, p-cholesterol, p-triglyceride, p-urate, p-parathyroid hormone and p-homocysteine.

At the other measurements and clinical controls the following blood samples are taken for analysis: b-hemoglobin, p-creatinine, p-urea, p-albumin, p-potassium and p-sodium.

The patient is placed supine on a bed in a quiet room and is resting for 15 minutes. A blood pressure is recorded by a mercury sphygmomanometer.

Data is recorded in the schedules attached as Annex IIA and IIB.

*Clinical controls*
Every 2^nd^ to 3^rd^ week the patient meets for blood pressure control and control of therapy. Blood sampling is carried out as described above. Study medication is adjusted.

*Chromium-EDTA clearance*There is a total of 2 study days for each patient on the department of clinical physiology, where the patient has a Cr-EDTA clearance performed. The days lies immediately before study start and after the termination of the study.
Intravenously injected 51 Cr-EDTA is excreted exclusively by glomerular filtration. Measurement of plasma concentration in one or more blood samples therefore allows calculation of the glomerular filtration rate.
At an estimated GFR <20 ml/min: 24 h clearance with blood sampling 5 and 24 hours after injection of 3.5 MBq of 51-Cr-EDTA.
At an estimated GFR> 20 ml/min: blood sampling after 180, 200, 220 and 240 min. after injection of 3.5 MBq 51-CR-EDTA.

5.13 STUDY MEDICATION

The study medication is the tablet Enalapril, an ACE inhibitor and the tablet Candesartan, which is an angiotensin receptor antagonist. Both drugs are approved drugs with existing SPC approved by the Medicines Agency. These will be available at the place of study, when the experiment is initiated. There is not decided any particular importer/manufacturer of this medication for this study.
Full treatment dose, respectively. Enalapril 20 mg and 16 mg candesartan once daily is the recommended maximum dose according Medicines catalog. Maximum dose is achieved as recommended after a titration period of 5-6 weeks. For combination treatment with both drugs, the study participant will already be in full dose treatment with one drug but will then slowly be uptitrated to full treatment dose of the other drug over 5-6 weeks. Dosing, route of administration, dosing frequency, titration and treatment period, are according to standard practice.
After the termination of the study period or withdrawal of the study, the participant returns to his usual medications.

The medicine will be delivered through Copenhagen County Central Pharmacy in ordinary medicine boxes. The medicine will be counted and packed by the investigator to each study participant for the scheduled period. Any handed medication will be recorded in the patient's Case Report Form, as well as any returned medication. There will also be taken account of receiving medication. The subjects will be thoroughly informed about the importance of taking the medication and that accounts will be kept with the medication for each patient. The frequent appearances of the patients are further helping to ensure a high patient compliance.

5.14 STATISTICS

Primary endpoint is PWV (brachial) and PWV (aortic) with a minimum clinically relevant difference of 1 m/s. Based on previous reproducibility (29) the standard deviation is 1.1 and 0.75 m/s for brachial and aortic PWV, respectively. The power of the t-test by comparing the two distributions is at n = 20 at 0.82 and 0.99 for brachial and aortic PWV, respectively.

A P-value less than 0.05 is considered significant.
Data from discontinued subjects can be assessed for each completed treatment period. Withdrawn participants are replaced by new subjects, so the size of patient population is achieved.
A professional statistician is associated with the project and has advised in creating the design. Data processing will be conducted under the guidance of the very same professional statistician.

**6. Quality control and quality assurance**General procedures for quality control and quality assurance will be followed in accordance with the ICH GCP Guidelines. Data will be monitored by the University GCP unit. In connection with the informed consent a permission is obtained from the study participants, that the research ethics committee, the Medicines Agency and the GCP unit can access information about their health. A copy of the proposed text is attached as Annex IIIa.

**7. Ethical considerations**
7.1 PROJECT IMPACT AND BENEFITS

The project will not have any consequences for the treatment of the individual patient. The intention is, that the project will lead to greater insight into the treatment of patients with chronic renal disease with drugs that block the renin-angiotensin system. This eventually allow for better intervention that may improve morbidity and mortality for patients with chronic nephropathy. Furthermore, the project contributes to an increased knowledge of applanationtonometry and its possibilities.

7.2 PROJECT RISKS AND DISADVANTAGES

1. Time consume
2. Discomfort of bloodsampling
3. Cr-EDTA clearance and thus ionized radiation

4. Adverse events associated with treatment

Removal of the maximum of 30 ml. of blood poses no risk.

The amount of ionizing radiation by a Cr-EDTA clearance is 0.01 mSv, and corresponds to one day of background radiation or radiation emitted by a conventional X-ray of an arm.

Applanationtonometry is noninvasive and poses no safety risk to the patient.

Adverse effects of ACE-I and AIIA are usually mild and transient.
When treated with ACE-I the following adverse effects can be seen: dry irritative cough, symptoms from the gastrointestinal tract such as nausea and diarrhea, and headache, fatigue, muscle cramps, and hyperkalemia. Angioneurotic edema, neutropenia and taste disorders are described. Hypotension. Renal impairment, which is usually reversible.
When treated with AIIA, the following adverse effects can be seen: dizziness, rarely orthostatic hypotension, rash, hyperkalemia and elevated liver enzymes, which normalize after discontinuation of therapy. Hypotension.
Contraindications for treatment of both drugs are renal artery stenosis or allergy towards the medicine.

It is ensured that fertile women are not pregnant when in the study in the form of a negative pregnancy test prior to enrollment in the trial. Moreover, it is a requirement for inclusion in the study that women of childbearing age use effective contraceptive methods. Safe contraception is due to the Medicines Agency one of the following methods: the pill, intrauterine device, depot gestagen injection, subdermal implantation, hormonal vaginal ring and transdermal patch. One of these methods must be used throughout the study period and for 1 week after the end of the study, which corresponds to 5 times the plasma half-life of the applied medication.

Recording and reporting of adverse events follow applicable laws. Referenced in the following text to the *Order of clinical trials of drugs in humans* defined types of side effect. Unexpected and serious suspected adverse reactions (Suspected Unexpected Serious Adverse Reactions, SUSAR) that are either fatal or life threatening will be reported to the Medicines Agency no later than 7 days after the sponsor-investigator has knowledge of them, and no later than 8 days after notification, the sponsor Investigators announce Medicines Agency all relevant information on follow-up. All other unexpected serious adverse reactions, will also be reported to the Medicines Agency no later than 15 days after the sponsor-investigator has knowledge of these. Serious, suspected adverse reactions (Serious adverse reaction, SAR) will be reported annually to the Medicines Authority. Serious adverse events and serious adverse events (Serious Adverse Events, SAEs) will be reported annually to the Scientific Ethical Committee. Adverse events and side effects will appear in the final report to the Medicines Authority of the study results.

If intolerable adverse effects, the participant will be withdrawn from the trial. The patient will then return to his usual medications and follow-up in the department of Nephrology, as long as required.

7.3 SUMMARY

The study patient population is a high risk group in terms of primarily cardiovascular morbidity and mortality. Based on several clinical controlled studies, blocking of the renin-angiotensin system have been shown to reduce this morbidity and mortality. Two of the three patient groups in the project will already be in treatment with either ACE-I or AIIA prior to the start of the study.
To minimize the risks of treatment, the patient population is carefully selected based on inclusion and exclusion criteria. Furthermore, there are frequent clinical and paraclinical control of the treatment in order to, at an early stage, observe any side effects or complications from the treatment. Finally, patients have the opportunity to contact a Nephrology doctor 24 hours a day if questions arise. Recording and reporting of adverse events follow applicable laws.

To sum, the advantages and disadvantages for the overall study group are assessed to balance.

The project will not be started until approval from the regional ethical committee, the Data Protection Agency and the Medicines Authority is obtained.
No trial participant will be included in the project before he has made a voluntary, written informed consent.
Patients who do not wish to participate in the project would not require justification, and the patient's participation or not should not affect the further monitoring or treatment of the patient.

The study is carried out in accordance with the Helsinki Declaration.

**8. Funding**

There are from Simon Fourner Hartmann Family Foundation awarded Dkr. 128,000 for the purchase of an applanationtonometer, Sphygmocor.
Kidney Association has contributed Dkr. 50,000.
Danish Society of Nephrology Research Foundation has contributed Dkr. 34.000
The Medical Research Foundation has contributed Dkr. 20,000
From Åse Bays Memorial Scholarship is awarded Dkr. 14.000 to the project.
Augustinus Foundation has contributed Dkr. 300.000
Hørslev Fund has contributed Dkr 400.000
Aase and Ejnar Danielsen's Foundation has contributed Dkr. 100.000.

Expenses:
Annual salaries for clinical assistant Dkr. 486.317,05
Medicine spending Dkr. 13.344,93
Total cost **Dkr. 499.661,98**

Salary costs are covered by own funds which are fully covered by the above statement of fund donations. Medical expenses are covered either by the Nephrology department, KAS Herlev, or from our own funds. Other operating expenses are covered by the Nephrology Department B, Copenhagen County Hospital in Herlev.
The project has no relation to the pharmaceutical industry.

**9. Patient information and consent form**Potential participants are patients with chronic nephropathy treated in Nephrology Department, Copenhagen County Hospital in Herlev. **GUIDELINES FOR SUBMISSION OF THE ORAL INFORMATION AND OBTAINING CONSENT.

Before the information interview**
By a usual consultation in our clinic the patient will be informed about the project. If the patient demonstrates interest an appointment is agreed upon time and place for an information interview. Before the information interview the patient will be pointed out that there is a request for participation in a scientific experiment. He is told, that he has the opportunity to bring a companion at the interview. Also reported that the patient at any time may withdraw from the project - without explanation - without influencing in any way the current or future treatment of the patient. He will at the initial interview be informed that he is entitled time for reflection after the information interview about the participation in the project.

**The information interview and reflection**
The information interview will take place in aquiet undisturbed physical conditions at the agreed time and place. The information is given by a physician in the project. The patient and any observer are informed verbally about the project. Then the patient is handed the written information with adequate time to read through. Subsequently any queries are answered. The patient is informed about the possibility of another interview with answering additional clarifying questions. Furthermore the patient is informed of the possibility of reflection before the patient signs the consent form. If the patient agrees to participate, he is asked to return the consent form within a week
after the information interview. The patient is at any time welcome to contact the project team doctors.

**Before the start of project**The patient will receive a signed copy of the consent form and meeting schedule for the measurements.

**10. Publication and reporting to authorities**

The results, both positive and negative, will be published in an internationally recognized medical journal.
The article is written by Marie Frimodt-Moller. Co-authors: Anne-Lise Kamper, Arne Høj Nielsen and Svend Strandgaard.

After completion, the sponsor-investigator will inform the Medicines Authority and the Scientific Ethical Committee of the termination of the study, within 90 days after the end of the study. The results of the study will as soon as possible thereafter be submitted to the Medicines Authority in accordance to the law on Medicinal Products § 24a, paragraph 2, no.4.
 **11. Insurance**

The investigator who is employed at Herlev University Hospital is covered by the hospital's accident and liability insurance. In case of patient death or injury is referred to the ‘Patient Insurance’ or law on ‘Compensation for drug injury’.
